# Importance of a Conserved Sequence Motif in Transmembrane Segment S3 for the Gating of Human TRPM8 and TRPM2

**DOI:** 10.1371/journal.pone.0049877

**Published:** 2012-11-21

**Authors:** Mathis Winking, Daniel C. Hoffmann, Cornelia Kühn, Ralf-Dieter Hilgers, Andreas Lückhoff, Frank J. P. Kühn

**Affiliations:** 1 Institute of Physiology, Medical Faculty, RWTH Aachen University, Aachen, Germany; 2 Department of Medical Statistics, RWTH Aachen University, Aachen, Germany; Monell Chemical Senses Center, United States of America

## Abstract

For mammalian TRPM8, the amino acid residues asparagine-799 and aspartate-802 are essential for the stimulation of the channel by the synthetic agonist icilin. Both residues belong to the short sequence motif N-x-x-D within the transmembrane segment S3 highly conserved in the entire superfamily of voltage-dependent cation channels, among them TRPM8. Moreover, they are also conserved in the closely related TRPM2 channel, which is essentially voltage-independent. To analyze the differential roles of the motif for the voltage-dependent and voltage-independent gating, we performed reciprocal replacements of the asparagine and aspartate within the S3 motif in both channels, following the proposed idea that specific electrostatic interactions with other domains take place during gating. Wild-type and mutant channels were heterologeously expressed in HEK-293 cells and channel function was analyzed by whole-cell patch-clamp analysis as well as by Ca^2+^-imaging. Additionally, the expression of the channels in the plasma membrane was tested by Western blot analysis, in part after biotinylation. For the mutations of TRPM8, responses to menthol were only compromised if also the expression of the glycosylated channel isoform was prevented. In contrast, responses to cold were consistently and significantly attenuated but not completely abolished. For TRPM2, surface expression was not significantly affected by any of the mutations but channel function was only retained in one variant. Remarkably, this was the variant of which the corresponding mutation in TRPM8 exerted the most negative effects both on channel function and expression. Furthermore, we performed an exchange of the inner pair of residues of the N-x-x-D motif between the two channels, which proved deleterious for the functional expression of TRPM8 but ineffective on TRPM2. In conclusion, the N-x-x-D motif plays specific roles in TRPM8 and TRPM2, reflecting different requirements for voltage-dependent and voltage-independent channel gating.

## Introduction

The channel structure of TRP channels and voltage-gated potassium channels is quite similar. Notably for TRPM8, the close structural similarity is associated with a related gating mechanism because a rudimentary voltage sensor element in the transmembrane segment S4 enables voltage-dependent activation of the channel [1]; [2]. In contrast to the classical voltage-dependent cation channels that exclusively respond to voltage changes across the plasma membrane, TRPM8 is additionally and more effectively stimulated by cold temperatures and various natural compounds from plants, e.g. menthol and eucalyptol [3]–[5]. The intensive search for the mechanism of channel activation by these chemical agonists revealed that a single tyrosine residue in transmembrane segment S2 is one important determinant for the interaction with menthol [6] and that several amino acid residues in the transmembrane segment S3 are critical for the sensitivity to the synthetic “super cooling agent” icilin [7]. In particular, the residue G805 within S3 is crucial because it is absent in the icilin-insensitive TRPM8 orthologs of birds. Two further amino acid residues, N799 and D802, were identified within S3 which are also critical for the interaction between TRPM8 and icilin [7]. However, the importance of these residues for the sensitivity of TRPM8 to menthol or cold has not been systematically analyzed so far.

The residues N799 and D802 are part of a short sequence motif, the so-called N-x-x-D motif (x-x stands for two hydrophobic amino acid residues), which is highly conserved in the S3 transmembrane segments not only of most voltage-dependent cation channels, but in some voltage-dependent TRP-channels and several voltage-independent TRP channels as well [8]. In a former study on voltage-gated Shaker K^+^-channels, a critical interaction between an aspartate in S3 (corresponding to D802 of TRPM8), and one of the basic residues of the S4 voltage sensor has already been demonstrated [9]. These data suggest that the S3 segment may bear greater and more general relevance for the function of TRPM8 than solely determining the sensitivity to a synthetic agonist, icilin.

Interestingly, TRPM2, the closest relative of TRPM8, contains the N-x-x-D motif within its S3 segment as well. However, TRPM2 does not respond to icilin or to any of the other stimuli of TRPM8, i.e. voltage, cold, and menthol. Not even after truncation of the C-terminal NUDT9H domain, after which TRPM2 becomes structurally closely similar to TRPM8, any responses to these stimuli were evoked [10].

The aim of the present study was to analyze the importance of the N-x-x-D motif for the gating of the channels TRPM8 and TRPM2 which are closely related in terms of structure but sensitive to quite different stimuli. Since electrostatic interactions of this motif with other transmembrane segments have been proposed [11], we swapped the position of the outer residues of the N-x-x-D motif or altered the hydrophobicity of the inner residues. We report strikingly differential effects on the responses to menthol and cold of TRPM8 and to ADP-ribose (ADPR) of TRPM2, reflecting the different modes of activation in spite of common essential structural elements.

## Materials and Methods

### Molecular Cloning

The cDNAs of human TRPM2 and TRPM8 were subcloned into pIRES-hrGFP-2a vector (Stratagene, La Jolla, CA, USA). Site-directed mutagenesis was performed using the QuikChange mutagenesis system (Stratagene). Defined oligonucleotides were obtained from MWG-Biotech (Ebersberg, Germany). Each point mutation was verified by DNA sequencing (MWG-Biotech). All procedures were performed in accordance to the respective manufacturer’s instructions, if not indicated otherwise.

### Cell Culture and Transfection

HEK-293 cells (German Collection of Microorganisms and Cell Cultures, Braunschweig, Germany) were seeded on poly-lysine-coated glass coverslips and grown for 24 h. Subsequently, the cDNAs of wild-type or mutated variants of TRPM2 and TRPM8 were transiently transfected into the HEK-cells using the FuGene 6 transfection reagent (Roche, Mannheim, Germany). As controls, HEK-cells were transfected with pIRES-hrGFP-2a vector alone. The transfected cells were maintained at 37°C and 5% CO_2_ in DMEM medium supplemented with 4 mM L-glutamine, 1 mM sodium pyruvate, 10% (v/v) fetal calf serum (Biochrome, Berlin, Germany). Patch-clamp or Ca^2+^-imaging experiments were carried out 24 h after transfection in cells visibly positive for EGFP. At least five independent transfections were used for each experimental group.

### Electrophysiology

Whole-cell recordings were performed using a EPC 9 amplifier equipped with a personal computer with Pulse 8.5 and × Chart software (HEKA, Lamprecht, Germany). The standard bath solution contained (in mM) 140 NaCl, 1.2 MgCl_2_, 1.2 CaCl_2_, 5 KCl, 10 HEPES, pH 7.4 (NaOH). For Na^+^ free solutions, Na^+^ was replaced by 150 mM N-methyl-D-glucamine (NMDG) and the titration was performed with HCl. The pipette solution contained (in mM) 145 CsCl, 8 NaCl, 2 MgCl_2_, 10 HEPES, pH 7.2 (CsOH) and the Ca^2+^ concentration was adjusted to either <10 nM (10 mM Cs-EGTA, no Ca^2+^ addition), or to 1 µM using 0.886 mM Ca^2+^ concentrations and 1 mM Cs-EGTA.

The Ca^2+^ concentration of the solutions were calculated using the *MAXC*-program: (http://www.stanford.edu/~cpatton/maxc.html). For the stimulation of TRPM2 currents, ADPR (100 mM stock solution in distilled water) was added to the intracellular solution yielding a final concentration of 0.1–1 mM. TRPM8 currents were induced with menthol (Sigma-Aldrich, 200 mM stock solution in DMSO) or icilin (*Cayman*, 30 mM stock solutions in DMSO) by application to the bath (final concentrations as indicated in the experiments). If not otherwise stated, the experiments were performed at room temperature (21°C) and the current-voltage relations were obtained during voltage ramps from –150 to +150 mV and back to −150 mV applied over 200 ms. The holding potential was −60 mV. The stimulation with cold was performed by adding ice-cold bath solution directly to the bath chamber during measurement. Thereby, the bath temperature was rapidly lowered to about 10°C (monitored with a digital thermometer). For analysis, maximal current amplitudes (pA) in a cell were divided by the cell capacitance (pF), a measure of the cell surface. The result is the current density (pA/pF).

### Intracellular Ca^2+^-imaging Experiments

For fluorescence imaging of [Ca^2+^]_i_ HEK-293 cells on poly-lysine-coated glass coverslips were loaded in standard bath solution containing membrane-permeable Fura-2 acetoxymethyl ester (1.5 ng/µl; Invitrogen) and pluronic acid (0,025%) for 20 min at 37°C. Fluorescence was alternately excited at 340 and 380 nm using the Polychrome IV monochromator (TILL Photonics). Emitted fluorescence was measured at 510 nm using a Sensicam (IMAGO). Fluorescence was corrected for background at each wavelength. Measurements were obtained at room temperature (21°C). Standard bath solution and stimulation with cold was identical as described for patch-clamp experiments. Stimulation with menthol was performed as described for patch-clamp experiments but the final concentration of menthol was increased to 300 µM.

### Analysis of Cell Surface Expression by Biotinylation and Western Blot

Membrane fractions of transiently transfected HEK-cells were prepared by differential centrifugation according to the protocol previously established by [12]. In brief, cells were grown to a confluence of 80–90% and transfection efficiency was determined by analysis of the rate of GFP-positive cells. Cells were washed with ice-cold PBS, subsequently collected by scraping and resuspended in 3 mL ice-cold lysis buffer (50 mM Tris-HCl, pH 7.5, 10 mM KCl, 1.5 mM MgCl_2_, 1 mM EDTA, 1 mM PMSF) containing a mammalian protease inhibitor cocktail (1∶100, Sigma-Aldrich). After incubation on an end-over-end stirrer for 30 minutes, cells were subjected to 3 freeze–thaw cycles and then were passed 10 times through a 26-gauge needle. To remove large cellular fragments, lysates were centrifuged at 4000 g. Supernatants were centrifuged at 20,000 g for 20 minutes and then at 100,000 g for 1 hour to obtain low-speed pellets representing the enriched plasma membrane fraction and high-speed pellets containing the bulk of intracellular membrane proteins.Subsequently, pellets were solubilized in ice-cold PBS containing 1% Triton X-100, 0.2% SDS, 1 mM PMSF and mammalian protease inhibitor cocktail. Protein concentration was determined by the Bradford-assay method using BSA as standard. Samples were normalized to their total protein content, supplemented with 4 × reducing sample buffer (Life Technologies), heated to 56°C for 15 minutes and subjected to SDS-PAGE and Western blot analysis.

As alternative to the differential centrifugation method, cell surface biotinylation assays using EZ-Link®Sulfo-NHS-SS-biotin were performed with the Pierce cell surface protein isolation kit according to the manufacturer’s instructions (Thermo Scientific). In brief, 36 h post-transfection, subconfluent HEK-293 cells (90%) expressing the corresponding TRPM2 variants were biotinylated and lysed in 500 µl of lysis buffer supplemented with mammalian protease inhibitor cocktail (1∶100, Sigma-Aldrich) and 1 mM PMSF. For each experimental condition, the same amount of protein (600 µg) was incubated with 500 µl of NeutrAvidin beads for 1 h at room temperature, after collecting a small aliquot of total cell lysate as input control and were eluted with SDS sample buffer (62.5 mM Tris-HCl, pH 6.8, 1% SDS, 10% glycerol, and 50 mM DTT) for immunoblot analysis. Cell membrane integrity was analysed by detection of β-actin as internal control (rabbit-anti-β-actin; Sigma-Aldrich; 1∶2000 in 5% BSA).

Samples were resolved on a 4–12% reducing Bis-Tris SDS-PAGE gel (NuPAGE, Life Technologies). After protein transfer to an activated PVDF-membrane and blocking (5% dry-milk in PBS), expression of wild-type or mutant hTRPM8 protein was determined by detection of immunoreactive products with a monoclonal rabbit-anti-human TRPM8 antibody (Epitomics; 1∶1000 in 5% dry-milk) directed against an extracellular domain or alternatively with a polyclonal rabbit-anti-human TRPM8 antibody (Abgent; 1∶500 in 5% dry-milk) directed against amino acids 1075–1104 of the C-terminus. Correspondingly, TRPM2 protein was detected with a polyclonal antibody (Abcam; 1∶1000 in 5% dry-milk) directed against amino acids 181–393. Bound primary antibody was detected using a mouse-anti-rabbit-HRP conjugated secondary antibody (DAKO A/S; 1∶1000 in 5% dry-milk). Detection was accomplished using the enhanced chemiluminescense Western blot detection system (ECL, Amersham Bioscience).

### Data Analysis and Statistics

Data are presented as means ± S.E.M. (standard error of the mean) for *n* cells. For evaluation of differential effects of menthol and cold on currents through various TRPM8 variants, a two way analysis of variance model was fitted to the logarithm of current densities, allowing for group differences with respect to channel variant (wild-type TRPM8, N799D, D802N, N799D+D802N), treatment (menthol or cold) as well as two way interactions. P-values less than 0.05 were considered significant. The data are presented in the Results and Discussion section with the following abbreviations: Ndf: nominator degrees of freedom; Ddf: denominator degrees of freedom; F: observed value of the F-statistic. All computations were performed with the SAS® software under Windows XP. Calcium imaging experiments were statistically evaluated with the Mann-Whitney-U Test. Bonferroni correction was applied when multiple comparisons were performed with the same control data.

## Results and Discussion

**Figure 1:**
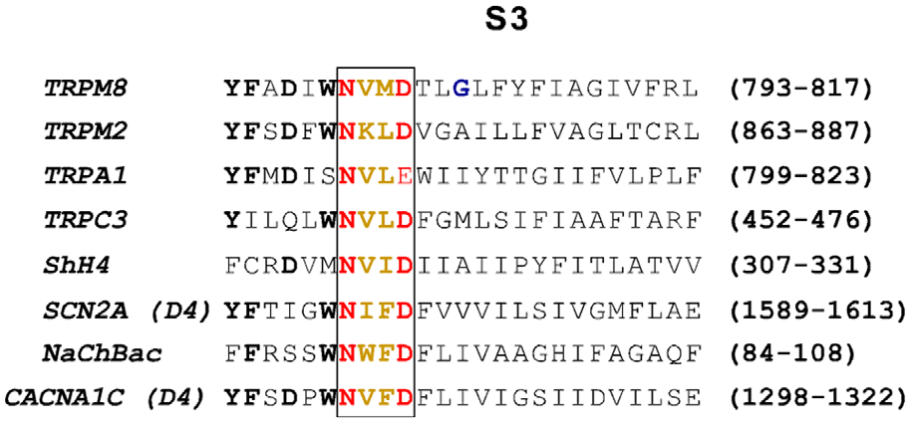
Conservation of a short sequence motif within transmembrane segment S3. Sequence comparison within the S3 region of selected voltage-gated as well as voltage-independent cation channels, including TRPM8 and TRPM2. The amino acid sequences are shown in single letter code. The highly conserved N-x-x-D motif is highlighted with the outer pair of amino acid residues labeled in red and the inner pair in orange. Further highly conserved amino acid residues upstream of the N-x-x-D-motif are given in bold letters. The glycine residue at position 805 in the sequence of human TRPM8 which is crucial for the icilin sensitivity of the channel is marked in blue. Accession numbers are as follows human TRPM8: Q7Z2W7; human TRPM2: O94759; human TRPA1: O75762; human TRPC3: Q13507, Shaker H4 (KCNAS_DROME): P08510; human sodium channel type 2 alpha subunit (SCN2A), domain 4: Q99250; voltage-gated sodium channel from Bacillus halodurans (NaChBac): Q9KCR8; human L-type calcium channel subunit alpha 1C (CACNA1C) domain 4: Q13936.

The N-x-x-D motif of transmembrane segment S3 starts at position 799 of the amino acid sequence of human TRPM8 as N-V-M-D, whereas it shows in human TRPM2 at the corresponding position 869 as N-K-L-D (**Fig. 1**). To analyze the importance of this sequence motif for gating by menthol and cold in TRPM8 as well as for gating by ADPR in TRPM2, we created mutants in which either the outer amino acid residues of the motif, i.e. asparagine and aspartate, were exchanged, or the inner pair of residues was swapped between TRPM8 and TRPM2. The resulting channel variants are summarized in **Table 1**. If either the outer or the inner residues act as specific interaction partner of adjacent channel domains, all these mutants should have distinct effects on channel function and possibly also on channel expression. The various channel variants were heterologously expressed in HEK-293 cells and functionally characterized with whole-cell patch-clamp analysis as well as with Ca^2+^-imaging. Moreover, the surface expression of the channel variants was tested with Western blot analysis of plasma membrane fractions of transiently transfected HEK-293 cells, using specific antibodies directed against hTRPM8 or hTRPM2.

**Table 1:** Variations of the N-x-x-D motif and corresponding nomenclature of channel variants examined in the study.

Mutation	sensor module sequence
wt-TRPM8	**N**-V-M-**D**
M8-N799D	**D**-V-M-**D**
M8-D802N	**N**-V-M-**N**
M8-N799D+D802N	**D**-V-M-**N**
M8-V800K+M801L	N-**K**-**L**-D
wt-TRPM2	**N**-K-L**-D**
M2-N869D	**D**-K-L-**D**
M2-D872N	**N**-K-L-**N**
M2-N869D+D872N	**D**-K-L-**N**
M2-K870V+L871M	N-**V**-**M**-D

### The Importance of the Outer Residues of the N-x-x-D Motif for the Gating of M8

**Figure 2:**
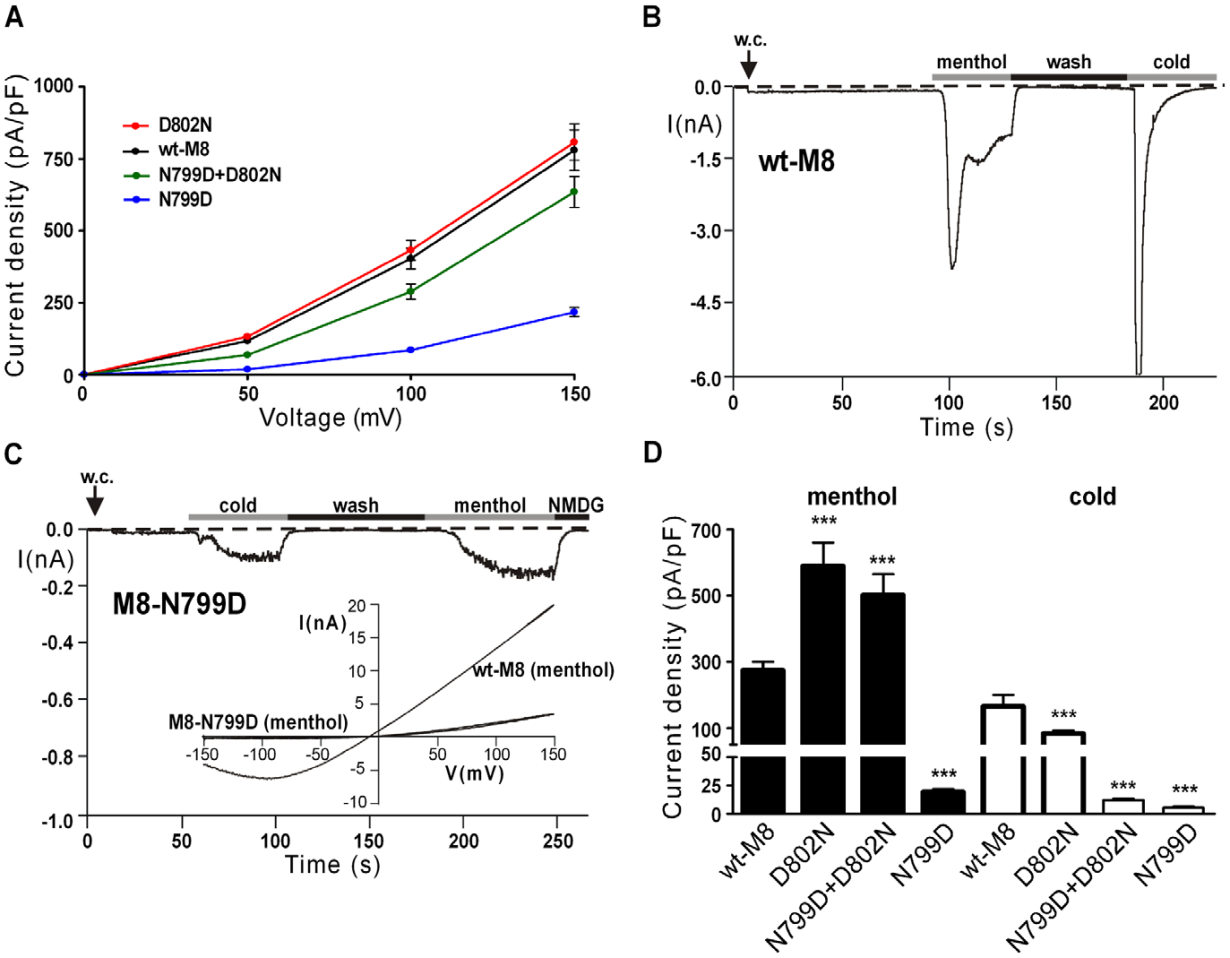
Current responses of TRPM8 variants to menthol and cold. The variants were wild-type, D802N, N799D, and N799D+D802N. (**A**), Current densities (representing mean ± S.E.M of 26–31 independent experiments) obtained at room temperature during voltage ramps between −150 mV and +150mV applied over 200 ms. Note that only the voltage range from 0 to +150 mV is shown because currents are sizeable exclusively in the outward direction (**B**), Whole-cell patch clamp measurement on HEK-293 cells expressing wild-type TRPM8 during stimulation with menthol (100 µM) or ice-cold bath solution as indicated by the horizontal bars. Between the two stimulations an intermediate wash-step with standard bath solution at room temperature was performed. The holding potential was −60 mV. (**C**), Similar experiment as shown in panel B on cells expressing the TRPM8 variant N799D. Note the different scaling of the ordinates in panels B and C. The inset shows the corresponding current-voltage relation of N799D in comparison to wild-type in the presence of menthol. (**D**), Mean inward current densities of the TRPM8 variants in response to menthol or cold obtained at a holding potential of −60 mV. Note that in double stimulation experiments only the data from the first stimulation were used for the statistical analysis. Each column represents mean ± S.E.M. of 10–16 independent experiments. Values of ***p<0.001 were considered extremely significant.

We first studied mutations of the outer residues of the N-x-x-D motif of TRPM8 (**Tab. 1**) Immediately after reaching the whole-cell configuration at room temperature, the current-voltage relation (IV-relation) of TRPM8 in the absence of a chemical stimulus is interpreted as activation of the channel induced by voltage and mild cooling. For wild-type TRPM8 (wt-M8) and all three TRPM8 mutants, **Figure 2A** shows the current densities at voltages between 0 and 150 mV, where the currents are outwardly-directed and most pronounced. In comparison to wild-type, the mutant N799D retained the characteristic outward rectification but the current densities were markedly reduced. The two other mutants exhibited comparable current densities to wt-M8.

In broad agreement with the data of a previous study [7], none of the mutants responded to icilin, even if the substance was applied at high concentrations (30–60 µM; data not shown). However, the mutants demonstrated a varying degree of sensitivity to cold and to menthol. Stimulation was either performed by superfusion of the cells with ice-cold bath solution or with a bath solution containing menthol (100–300 µM). **Figures 2B and 2C** show representative recordings where both stimuli were consecutively applied but were separated by a wash step with standard bath solution at room temperature. For statistical analysis such as shown in **Figure 2D** data either from single stimulation experiments or from the first stimulation of double stimulation experiments were exclusively used. In this way, inadvertent effects induced by desensitization or tachyphylaxis were avoided.

Cells which express the mutant N799D responded to menthol (100 µM) with currents, showing a characteristic outward rectification in the IV relation but amplitudes one magnitude below that of wt-M8 (**inset of** **Fig. 2C** **,** **Figs. 2B–2D**). Only marginal currents were induced in N799D by superfusion with ice-cold bath solution, if compared to wt-M8 (**Figs. 2B–2D**).

**Figure 3:**
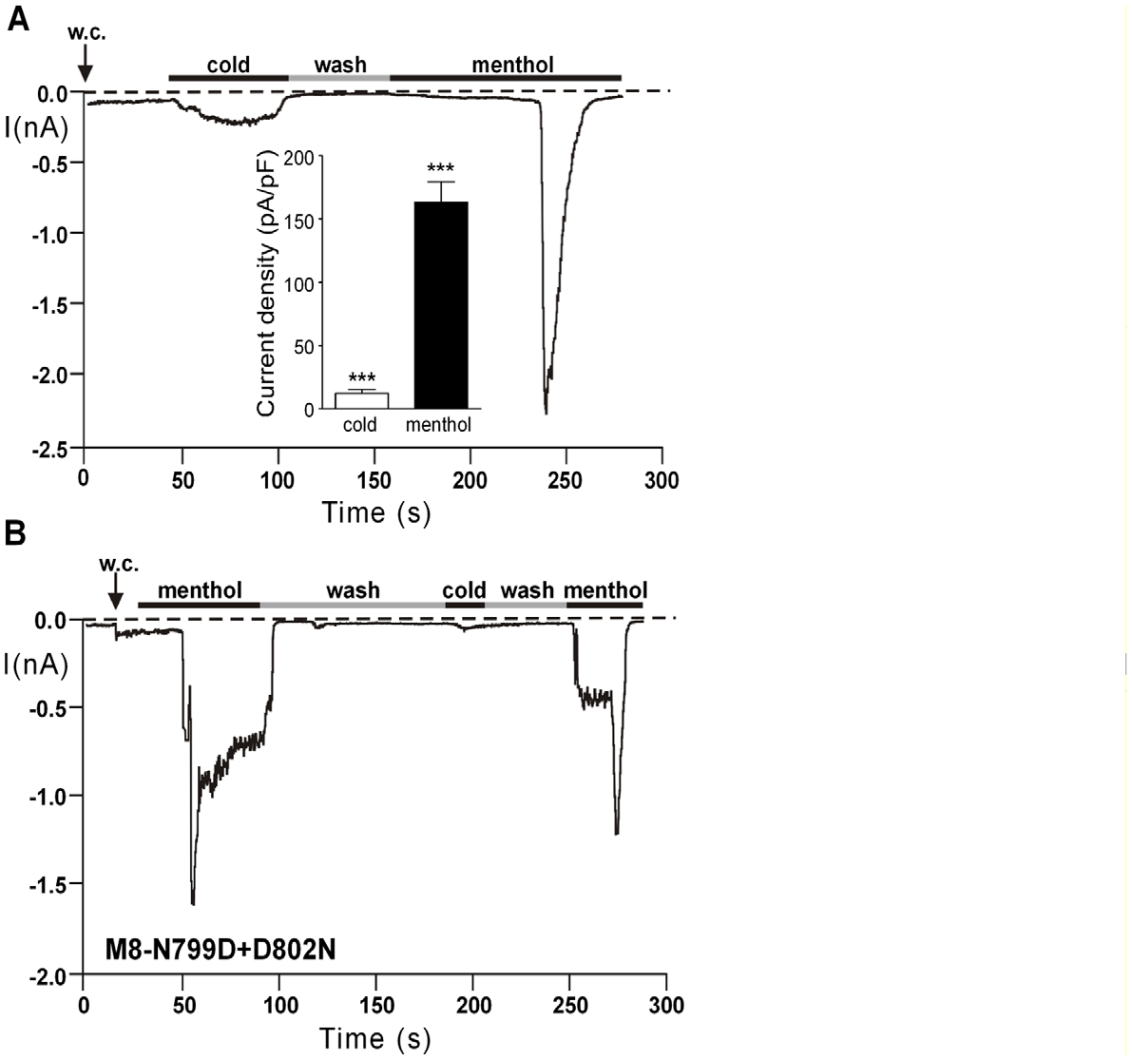
Differential effects of the mutation M8-N799D+D802N on the responses to cold and menthol. (A) Consecutive stimulation of a cell expressing M8-N799D+D802N, initially with cold and then with menthol (100 µM). Cells were exposed to room temperature prior to the exposure to menthol. The inset shows the summarized data with each column representing mean ± S.E.M. of 6 independent experiments. The difference between the values was highly significant (***p<0.001). (B) Consecutive stimulation with the two stimuli in the reversed order. The response to cold was even weaker than in panel A, due to desensitization that also weakened a further reaction to menthol. Note that for each experiment only the data from the first stimulation were used for the statistical analysis.

The two other mutants N799D+D802N and D802N showed increased currents in response to menthol, as compared to wild-type. In contrast, the sensitivity towards cold was reduced (**Fig. 2D**). An extended statistical analysis was performed to clarify whether the mutations affected the responses to menthol and to cold in a differential manner. After deletion of one observation (out of 95) (that would have influenced the parameter estimate too strongly), model diagnostic was performed. There were significant differences between channel variants (F = 153.34, ndf 3, ddf 85, p<0.0001), the treatments (F = 303.97, ndf 1, ddf 85, p<0.0001) and the two way interaction (F = 40.05, ndf 3, ddf 85, p<0.0001). The relative strength of temperature to menthol responses in wt-M8 did not significantly differ to that in N799D, but was significantly smaller in D802N (t = −4.03, df = 85, p = 0.0001) as well as in N799D+D802N (t = −10.26, df = 85, p<0.0001). The same finding is illustrated in experiments when consecutive stimulations (first with cold and then with menthol) were performed on M8-N799D+D802N (Fig 3A). Normally, desensitization prevents meaningful comparisons between consecutive stimulations of TRPM8 but in this case, the second response (i.e. that to menthol) was considerably (more than 15-fold) and significantly larger (n = 6) than the first one (i.e. that to cold). When the stimulation was performed in reversed order (first menthol, then cold, followed again by menthol), cold again evoked a much weaker reaction than menthol (Fig. 3B). Moreover, it is demonstrated that desensitization does not account for the weak reaction to cold because a further stimulation with menthol evoked currents that were considerably more pronounced than during exposure to cold, although smaller in amplitude than during the first menthol stimulation (Fig. 3B).

Thus, both the mutations D802N and N799D+D802N affected the sensitivity to cold much stronger than the sensitivity to menthol, even though cold reactions were still present, in contrast to responses to icilin.

**Figure 4:**
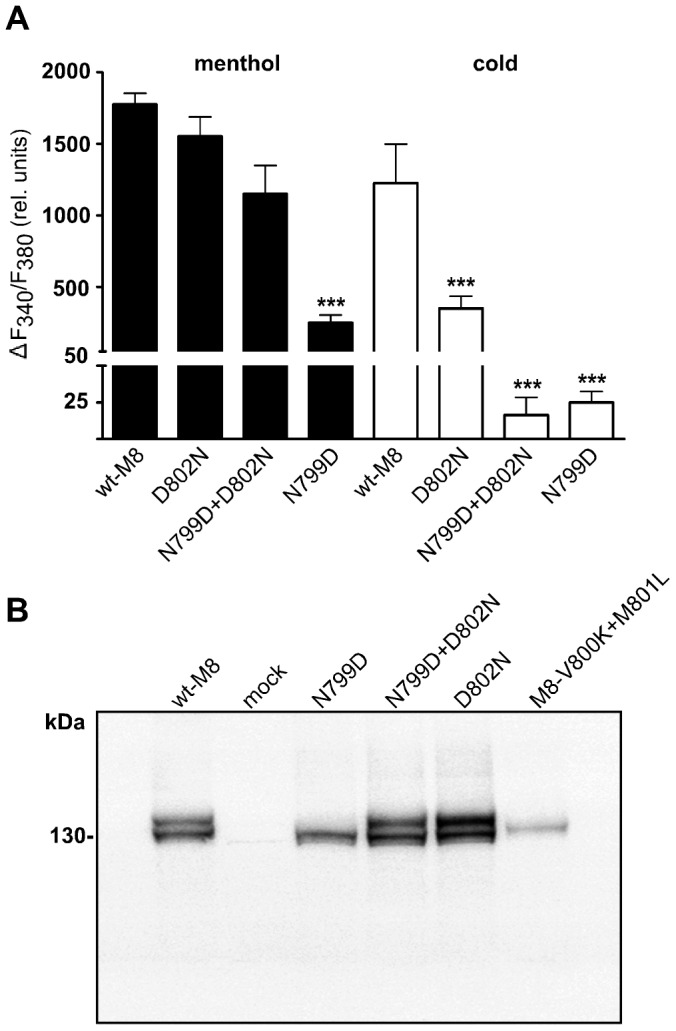
Activity of TRPM8 variants in intracellular Ca^2+^ measurements and Western blot of plasma membrane preparations. (**A**), Maximum increase in the F_340_/F_380_ ratio of each variant in response to 300 µM menthol or ice-cold bath solution. In double stimulation experiments only the data from the first stimulation were used for statistical analysis. The miniscule increases in fluorescence observed in mock-transfected controls are subtracted. Each column represents mean ± S.E.M. of 8–17 independent experiments. Significant differences to control are indicated with asterisks. Values of ***p<0.001 were considered extremely significant. (**B**), Western blot of TRPM8 protein on plasma membrane fractions prepared by the differential centrifugation method (“low speed fraction” ref. to Material and Methods) of HEK-293 cells expressing various TRPM8 variants. Note the dual bands, shown in previous studies to indicate the glycosylated and non-glycosylated channel protein. The glycosylated form is absent in the weakly functional channel variant N799D. As negative control a plasma membrane fraction of mock-transfected HEK-293cells is included.

The current measurements were confirmed in the more functional Ca^2+^ imaging experiments (**Fig. 4A**). Here, the stimulation was performed either by superfusion of the cells with ice-cold bath solution or by application of high concentrations of menthol (300 µM). Again, responses to menthol were strongly and significantly reduced exclusively for N799D, whereas the relative strength of the cold responses was significantly attenuated in all mutated variants.

The Western blot signals of enriched plasma membrane fractions obtained by the differential centrifugation method (“low speed fraction”, ref. to Material and Methods) from cells expressing wt-TRPM8 show a characteristic double band in the range of 130 kDa (**Fig. 4B**). According to the results obtained from other groups [13]–[15], the higher molecular mass signal is likely to be due to glycosylation of the TRPM8 channel protein and strong evidence for surface expression. The variants D802N and N799D+D802N showed no qualitative differences to wt-M8. Slightly increased signals for the upper or lower band (e.g. the upper band of the variant D802N in **Fig. 4B**) were not consistent in independent preparations and cannot be associated to a certain channel variant. In contrast to the other variants and in agreement with the functional data, the variant N799D consistently produced considerably weaker signals and the glycosylated band was not detectable. The same results of the Western blot analysis were obtained in independent experiments using two different types of primary antibodies, as described in Material and Methods (not shown). Thus, the expression data suggest that N799D may have compromised channel function due to diminished expression, especially of the glycosylated form, whereas the differentially modified responses to menthol and cold of the other variants cannot be solely explained by expression.

These data demonstrate that the N-x-x-D motif in fact is essential for the sensitivity of TRPM8 to icilin but is also critical for the responses to menthol and to cold, and therefore experimentally support the recently proposed model [11] based on molecular dynamic simulations, strongly suggesting that S3 is essential for the activation of TRPM8 by menthol.

### The Importance of the Outer Residues of the N-x-x-D Motif for the Gating of M2

**Figure 5:**
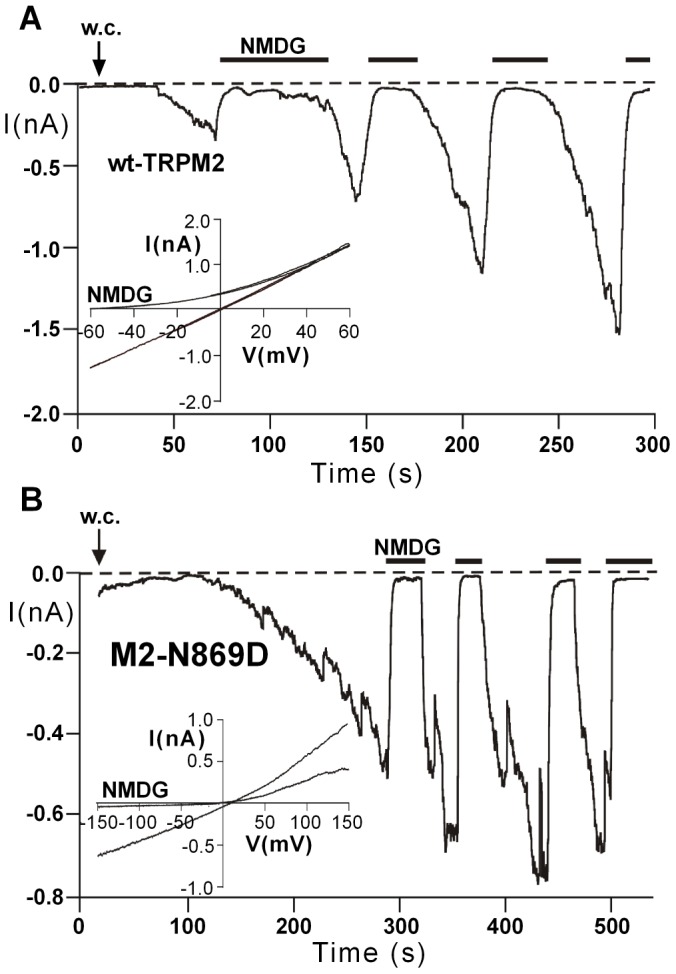
Current responses of the closely related TRPM2 channel and its variant N869D. Whole-cell patch clamp measurements on HEK-293 cells expressing wild-type TRPM2 (**A**) or TRPM2-variant N869D (**B**). Stimulation was performed with ADPR (600 µM in the absence of intracellular Ca^2+^) infused into the cell through the patch pipette. The holding potential was −60 mV. Insets show the current-voltage relations obtained during voltage ramps within the indicated voltage ranges. Inward currents were repeatedly blocked with NMDG. The TRPM2 variant N869D is analogous to the virtually non-functional TRPM8 variant N799D.

**Figure 6:**
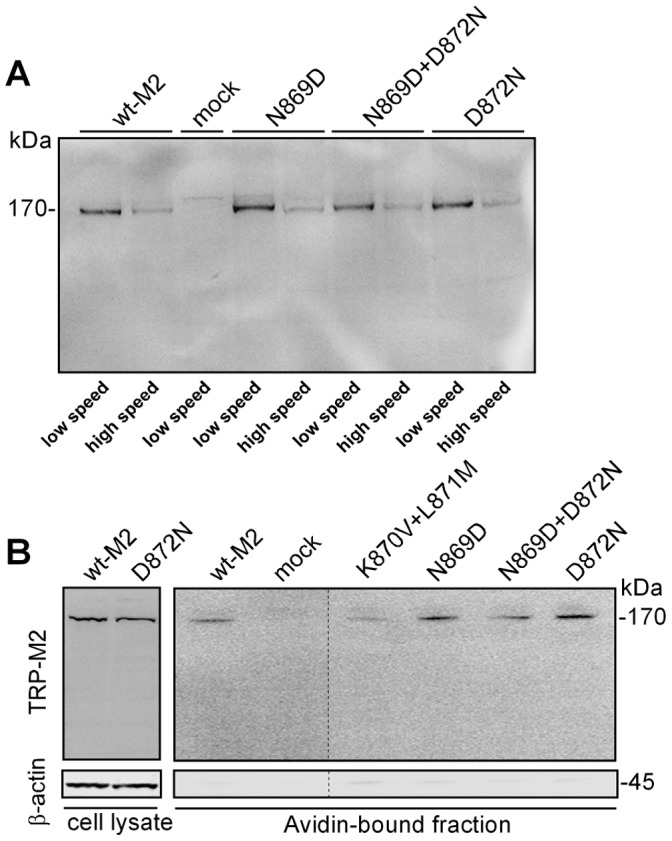
Cell surface expression of TRPM2 variants. (A) Western blots were performed on two different membrane fractions of HEK-293 cells transfected with a TRPM2 variant and probed with a commercially available anti-TRPM2 antibody. Mock-transfected cells were used as negative control. Membrane fractions were obtained either with high or low speed centrifugation (20,000 g vs. 100,000 g) of whole-cell lysates. (B) Alternatively, cell surface expression of TRPM2 channel variants as well as mock-control was monitored by biotinylation assays. Eluted samples were immunoblotted and detected by the same anti-TRPM2 antibody as used in A (upper right panel). Total TRPM2 expression was assessed by loading unpurified cell lysate (upper left panel). Membrane integrity and exclusive biotin-labeling of cell surface proteins was verified by re-incubation of the blot with an anti-β-actin antibody and loss of β-actin signal in the NeurAvidin-bound fraction (lower panel). Two independent experiments gave similar results.

Remarkably, TRPM2 as the closest relative of TRPM8 within the TRP-family also contains the conserved N-x-x-D motif of S3 (**Fig. 1**), offering the unique opportunity to test the importance of the motif not only for the voltage-dependent gating of TRPM8 but also for the voltage-independent gating of TRPM2. Hence, we constructed the same set of mutants for TRPM2 as for TRPM8, in particular N869D, D872N, and N869D+D872N (**Tab. 1**). Stimulation was performed with an intracellular solution containing ADPR (0.1 to 1 mM) in the absence or presence of Ca^2+^ (<10 nM or 1 µM). The mutation N869D (the analogue of which had almost abrogated channel expression and function in TRPM8) produced current responses in TRPM2 that were closely similar to wild-type (n = 16) because currents developed rapidly to amplitudes exceeding 0.5 nA (holding potential −60 mV) before they were blocked with NMDG to prevent damage to the cells (**Fig. 5**). In contrast, neither D872N (n = 25) nor N869D+D872N (n = 27) could be stimulated, even if high intracellular concentrations of ADPR and Ca^2+^ were used (data not shown). To exclude that the virtual loss of function observed for the two TRPM2 variants D872N and N869D+D872N was due to compromised expression in the plasma membrane, two experimental approaches were followed. Initially, Western blots were performed on two different fractions of cell membrane preparations, one obtained by low speed (20,000 g) centrifugation yielding enrichment of the plasma membrane proteins, and the other obtained by high-speed centrifugation (100,000 g) containing the bulk of intracellular membrane proteins. There was a weak band well above 170 kDa in mock-transfected cells which appeared in all other lanes as well and should be considered non-specific for TRPM2. The specific band for TRPM2 appeared approximately at 170 kDa and was clearly enriched in the plasma membrane fraction for all TRPM2 variants (Fig. 6A). Since the Western blots of TRPM2 and its variants do not show evidence for glycosylation as observed for TRPM8, we performed biotinylation assays as second and more rigorous test for cell surface expression of the TRPM2 channel proteins (Fig. 6B). Again, there were no obvious differences in the surface expression between wt-TRPM2, N869D, D872N, and N869D+D872N. All variants uniformly show a single band at approximately 170 kDa, whereas this signal was undetectable in mock-transfected HEK-cells (**Fig. 6B**).

The Western blots show that the loss-off-function phenotype of the TRPM2 channel variants D872N and N869D+D872N cannot be explained with a failure of expression, in contrast to the results obtained with the TRPM8 variant N799D. The data demonstrate that the aspartate residue of the N-x-x-D motif, which is less important for TRPM8, is absolutely required on its correct position for the proper function of TRPM2. Thus, the N-x-x-D motif is critical for TRPM2 as well but in a remarkably different way that probably reflects the different modes of gating in TRPM2 and TRPM8.

### The Importance of the Inner Residues of the N-x-x-D Motif for the Gating of TRPM2 and TRPM8

**Figure 7:**
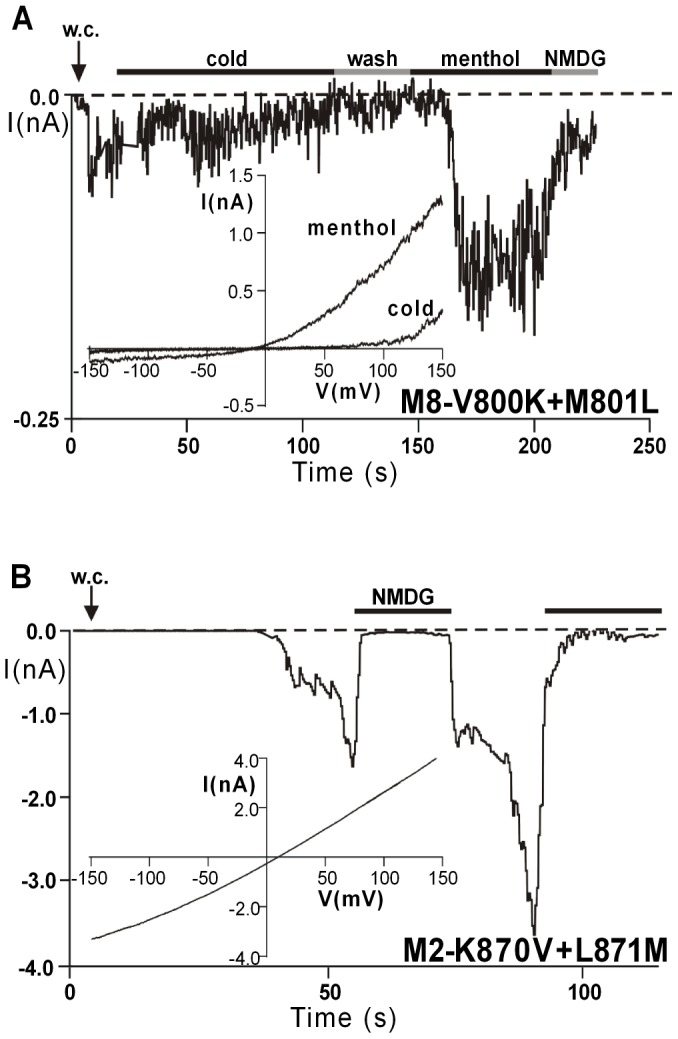
Importance of the inner residues within the N-x-x-D motif for the activity of TRPM8 and TRPM2. Whole-cell patch clamp measurements on HEK-293 cells expressing variants of TRPM2 and TRPM8 where the inner pair of residues of the N-x-x-D motif is reciprocally exchanged. (**A**), TRPM8 variant V800K+M801L first stimulated with ice-cold bath solution and after an intermediate wash-step with standard bath solution stimulated with 100 µM menthol (as indicated by horizontal bars). (**B**), TRPM2 variant K870V+L871M stimulated by infusion of 600 µM ADPR into the cell through the patch pipette and in the absence of intracellular Ca^2+^. Insets show the corresponding current-voltage relations during stimulation.

Kumanovics and coworkers [8] have identified the N-x-x-D-motif, where the x-x stands for two hydrophobic amino acid residues, as a typical S3-sequence for most of the classical voltage-gated cation channels (**Fig. 1**). In almost all of the known orthologs of TRPM8, which essentially represents a voltage-gated ion channel [1]; [2], these hydrophobic residues invariably are valine and methionine, whereas in the voltage-insensitive TRPM2 channel, the clearly less hydrophobic sequence lysine and leucine is conserved between many species. In order to test the functional relevance of these inner residues, we performed an exchange of them between TRPM2 and TRPM8 and then analyzed the consequences in whole-cell patch-clamp experiments of HEK-293 cells expressing the corresponding channel mutants. The TRPM8 mutant V800K+M801L that contains the sensor module sequence of TRPM2, i.e. N-K-L-D (**Tab. 1**), displayed a marked decrease of the sensitivity to menthol or to cold temperatures **(** **Fig. 7A**). Additionally, the glycosylated channel isoform was absent in the Western blot analysis (**Fig. 4B** **)**. Thus, the phenotype of the mutant V800K+M801L is similar to that of the mutant N799D **(** **Fig. 2C** **)**. In contrast, the TRPM2 mutant K870V+L871M that contains the sensor module sequence of TRPM8, i.e. N-V-M-D (**Tab. 1**), produced current responses that were comparable to those typically obtained with wt-TRPM2 **(** **Figs. 5A** **and** **7B**). In the light of these functional results, the slightly reduced brightness of the signal in the biotinylation assays (Fig. 6B) should not be overinterpreted.

These data suggest that the inner pair of hydrophobic amino acid residues of the S3 sensor module plays a crucial role for the channel function of TRPM8 but is less important for the gating of TRPM2. Possibly, hydrophobic residues stabilize electrostatic interactions between menthol and icilin on one hand and the transmembrane segments S2 and S3 on the other hand [16].

Taken together, the data demonstrate the importance of the N-x-x-D motif in the S3 segment for both, the voltage-dependent activation of TRPM8 that is essentially supported by ligands and cold, and the voltage-independent activation of TRPM2 by ADPR. In the case of TRPM8, the absolute requirement of the motif for activation by icilin has previously been demonstrated [7]. The present data demonstrate that mutations in this region may not only affect the activation by menthol and cold but additionally even the expression in the plasma membrane. Comparable findings have been reported for Shaker K^+^-channels in which the same highly conserved motif is present [9]; [17] (**Tab. 1**).

Strongly reduced channel function was found when the glycosylated isoform was absent in Western blots (e.g. channel variants M8-N799D and M8-V800K+M801L). The aspartate residue at position 799 cannot be a major glycosylation site of TRPM8 as it is located within the plasma membrane or at the intracellular face of the cell membrane according to standard structure models of TRPM8 [13]. This assumption is supported by our finding that the variant N799D+D802N displayed a wild-type-like Western blot pattern. Therefore, the lack of glycosylation is interpreted to indicate problems with expression in general, possibly the correct channel assembly or insertion into the plasma membrane. As shown for voltage-dependent potassium channels in the study of Zhang and coworkers [17], it is the combination of hydrophobic and electrostatic forces involving S2, S3, and S4 that controls the proper membrane insertion of the channel protein. Membrane expression was affected by mutations (i.e. N799D and V800K+M801L) exclusively in TRPM8 which is similarly voltage-dependent as Shaker K^+^-channels. In contrast, the expression of the voltage-independent TRPM2 was not altered by any tested mutations but only the function. Remarkably, the mutations D872N and N869D+D872N abolished function of TRPM2, in distinct contrast to N869D which in turn was (as the corresponding mutation N799D) most deleterious on TRPM8. Here it even diminished the response to menthol. In general, responses to menthol were only compromised when the expression of the glycosylated isoform of the channel protein was diminished as well. Therefore, the role of individual residues in the N-x-x-D motif cannot be attributed to the gating process initiated by menthol; rather, a more general function that already affects membrane insertion is strongly suggested by the Western blots that revealed dramatic effects of some single amino acid exchanges. For the gating by cold, however, a specific and expression-independent perturbation has been demonstrated in some mutations (e.g. M8-D802N, M8-N799D+D802N). This is most evident in the double mutant M8-N799D+D802N (Figs. 2D and 3). It may be hypothesized that responses to cold involve an especially delicate interaction with the plasma membrane, in contrast to the interaction with menthol, for which obviously less stringent structural requirements exist in the S3 domain. The removal or addition of negative charges in S3 possibly disturbs the interaction with the equally negatively charged phosphatidylinositol 4,5-bisphosphate (PIP2), which has been demonstrated to be crucial for the gating of TRPM8 [18]. In line with this hypothesis, no such negative effects were elicited by changing the charge distribution in the S3 segment of TRPM2 of which the gating is less essentially dependent on PIP2 [19].

In conclusion, the differential consequences of the tested mutations within S3 of TRPM8 and TRPM2 are likely to reflect a differential role exerted by the N-x-x-D motif in the various gating modes of TRPM8 and TRPM2. The present results may be the basis for future studies that will address intramolecular interactions specific for each gating process and may identify possible interaction partners of S3.
